# A Photosensitizer-Loaded Polydopamine Nanomedicine Agent for Synergistic Photodynamic and Photothermal Therapy

**DOI:** 10.3390/molecules28155874

**Published:** 2023-08-04

**Authors:** Shufeng Yan, Luying Dong, Ziyun Hu, Yucheng Zhang, Wei Xu, Jianhong Xing, Juncheng Zhang

**Affiliations:** 1 Medical Plant Exploitation and Utilization Engineering Research Center of Fujian Province, Sanming University, Sanming 365004, China; 2 School of Resource and Chemical Engineering, Sanming University, Sanming 365004, China

**Keywords:** nanomedicine, photodynamic therapy, photothermal therapy, photosensitizer, synergistic therapy

## Abstract

Photodynamic therapy (PDT) and photothermal therapy (PTT) have emerged as promising non-invasive approaches to cancer treatment. However, the development of multifunctional nanomedicines is necessary to enhance these approaches’ effectiveness and safety. In this study, we investigated a polydopamine-based nanoparticle (PDA-ZnPc^+^ Nps) loaded with the efficient photosensitizer ZnPc(4TAP)^12+^ (ZnPc^+^) through in vitro and in vivo experiments to achieve synergistic PDT and PTT. Our results demonstrated that PDA-ZnPc^+^ Nps exhibited remarkable efficacy due to its ability to generate reactive oxygen species (ROS), induce photothermal effects, and promote apoptosis in cancer cells. Moreover, in both MCF-7 cells and MCF-7 tumor-bearing mice, the combined PDT/PTT treatment with PDA-ZnPc^+^ Nps led to synergistic effects. Subcellular localization analysis revealed a high accumulation of ZnPc^+^ in the cytoplasm of cancer cells, resulting in cellular disruption and vacuolation following synergistic PDT/PTT. Furthermore, PDA-ZnPc^+^ Nps exhibited significant antitumor effects without causing evident systemic damage in vivo, enabling the use of lower doses of photosensitizer and ensuring safer treatment. Our study not only highlights the potential of PDA-ZnPc^+^ Nps as a dual-functional anticancer agent combining PDA and PTT but also offers a strategy for mitigating the side effects associated with clinical photosensitizers, particularly dark toxicity.

## 1. Introduction

Over the past few decades, significant progress has been made in cancer treatment methods and drug innovation, leading to improved quality of life for patients [1,2,3]. However, cancer remains one of the most devastating diseases worldwide, with a projected 1,958,310 new cancer cases and 609,820 cancer-related deaths in the United States in 2023 [4,5]. Therefore, there is an urgent need for continued research into cancer treatment. In this regard, combining various treatment modalities, particularly photodynamic therapy (PDT) and photothermal therapy (PTT), with anticancer drugs and nanomedicines has garnered considerable attention from researchers [6,7,8,9].

Nanomedicine, a field that has been extensively studied, has shown promising applications in antitumor therapy [10,11]. The notable advantage of nanomaterials lies in their ability to integrate the functions of anticancer drugs and enhance therapeutic efficacy through the enhanced permeability and retention (EPR) effect [12,13,14]. By exploiting the EPR effect, nanomaterials can facilitate the targeted delivery of nanosized anticancer drugs to tumor tissues [15,16]. Among the various types of nanomaterials, polydopamine (PDA) has garnered significant interest due to its excellent biocompatibility, drug-loading capabilities, and efficient photothermal conversion capacity [17,18,19]. Consequently, PDA has been widely investigated with respect to its potential in photothermal therapy, utilizing its ability to induce significant photothermal effects in order to eliminate tumor cells [20,21].

Photodynamic therapy, an approved innovative therapeutic approach for cancer treatment, offers improved applicability [22,23,24]. The efficacy of photodynamic therapy relies on the production of reactive oxygen species (ROS) by the photosensitizer, which can directly react with biological substrates, leading to toxicity and subsequent cancer cell death [25,26,27]. Zinc phthalocyanine (ZnPc) has been proven to be a type of photosensitizer with favorable photodynamic effects [28,29,30]. Despite the acceptance of PDT as a treatment modality, it still has limitations, particularly the occurrence of dark toxicity at higher concentrations, which hinders its widespread application [31,32]. Therefore, there is an urgent need to explore therapeutic strategies that enable the use of lower doses of photosensitizers while significantly improving the efficacy of anti-tumor therapy.

**Figure 1:**
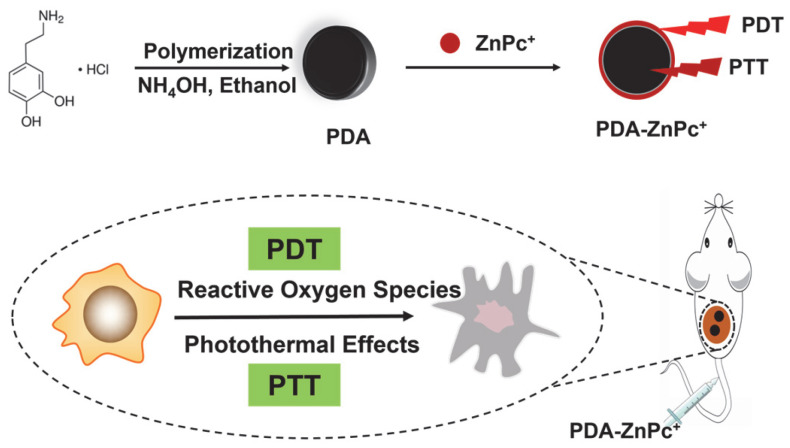
The synthetic and schematic diagram of PDA-ZnPc^+^ Nps in synergistic PDA and PTT.

In order to enhance anti-tumor efficacy, we propose a synergistic anticancer strategy that combines photodynamic therapy (PDT) with photothermal therapy (PTT) using a photosensitizer-loaded nanomaterial PDA-ZnPc^+^ Nps. As depicted in Figure 1, we have developed a polydopamine (PDA)-based nanoparticle that is loaded with the photosensitizer ZnPc(4TAP)^12+^ (ZnPc^+^) to achieve synergistic PDT and PTT. ZnPc^+^, a water-soluble photosensitizer synthesized from ZnPc and 2,4,6-tris(N,N-dimethylaminomethyl) phenoxy (TAP), has proven to be an effective anticancer photosensitizer [33,34]. In addition to the photothermal effects of PDA, the PDA-ZnPc^+^ Nps also exhibit PDT-mediated anticancer actions, enabling the direct killing of tumor cells. The combined therapy of PDA and PTT synergistically improves the efficacy of cancer treatment while reducing the side effects associated with photosensitizers. In this study, we comprehensively evaluate PDA-ZnPc^+^ Nps both in vitro and in vivo, including with respect to their synthesis and characterization, and perform an assessment of ROS generation and photothermal effects, apoptosis detection, and PDT and PTT efficacies in MCF-7 cells and MCF-7 tumor-bearing mice. Our findings demonstrate that this dual-functional nanomedicine, which is capable of achieving synergistic PDA and PTT, holds significant potential with respect to combination therapy for cancer treatment.

## 2. Results

### 2.1. Synthesis and Characterization of PDA-ZnPc^+^ Nps

The synthesis of PDA-ZnPc^+^ Nps is depicted in Figure 1. Briefly, PDA nanoparticles and ZnPc^+^ (Figure S1) were separately synthesized. PDA nanoparticles were then mixed with excess ZnPc^+^ and stirred for 2 h in the dark. The resulting mixture was centrifuged and washed with DI water multiple times until the supernatant became colorless, yielding the PDA-ZnPc^+^ Nps. ZnPc^+^, possessing 12 positive charges, could electrostatically attach to the negatively charged surface of PDA.

**Figure 2:**
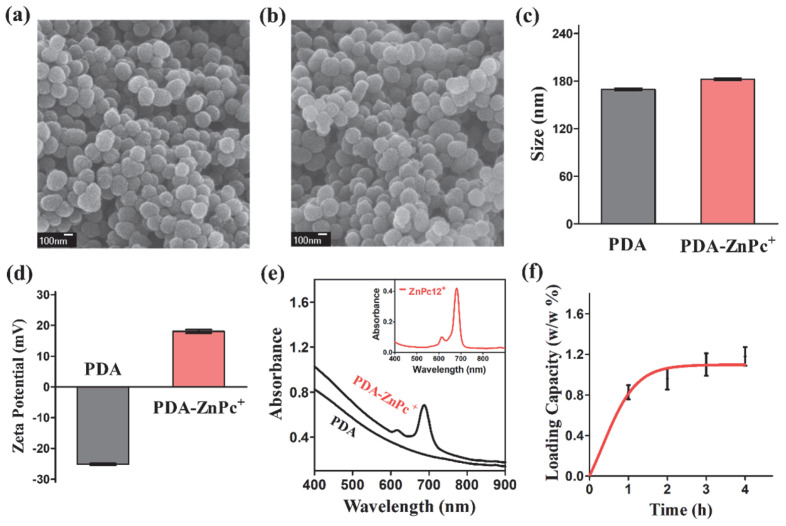
(**a**) SEM image of PDA Nps. (**b**) SEM image of PDA-ZnPc^+^ Nps. (**c**) Size distributions of PDA, and PDA-ZnPc^+^ Nps in water measured with DLS. (**d**) Zeta potentials of PDA and PDA- ZnPc^+^ Nps measured via DLS. (**e**) UV−Vis absorption spectra of PDA and PDA-ZnPc^+^ Nps. Inset: UV−Vis absorption spectrum of ZnPc^+^. (**f**) Quantifications of ZnPc^+^ loading at different incubation times.

The scanning electron microscopy (SEM) images of PDA Nps and PDA-ZnPc^+^ Nps are presented in Figure 2a and Figure 2b, respectively. The obtained PDA-ZnPc^+^ Nps displayed a uniform spherical morphology with a mean size of approximately 170 nm, similar to that of PDA Nps. Both PDA and PDA-ZnPc^+^ Nps exhibited good dispersibility and uniformity. The size distributions and zeta potentials of PDA and PDA-ZnPc^+^ Nps were further characterized using dynamic light scattering (DLS). The results showed that the average hydrodynamic diameters of PDA-ZnPc^+^ Nps were comparable to those of PDA Nps (Figure 2c), while their zeta potentials exhibited significant changes (Figure 2d), indicating the successful loading of ZnPc^+^ onto the PDA Nps. To confirm the successful synthesis of PDA-ZnPc^+^ Nps, the UV-vis absorption spectra of PDA and PDA-ZnPc^+^ Nps were examined. The results demonstrated distinct differences between the absorption spectra of the PDA and PDA-ZnPc^+^ Nps (Figure 2e). It is worth noting that a ZnPc^+^ characteristic peak (690 nm) was exhibited in the absorption spectrum of the PDA-ZnPc^+^ Nps, thus confirming the formation of PDA-ZnPc^+^ Nps. Then, a quantitative analysis of ZnPc^+^ loading (Figure 2f) revealed that the content of ZnPc^+^ attached to the PDA-ZnPc^+^ Nps was 1.18% (*w*/*w*), indicating a significant interaction between PDA and ZnPc^+^. Furthermore, negligible changes in size distribution were observed for the PDA-ZnPc^+^ Nps over a 7-day period (Figure S2), indicating their stability. Additionally, the PDA-ZnPc^+^ Nps exhibited high stability without noticeable agglomeration in water, saline, and PBS over 24 h (Figure S3).

### 2.2. ROS and Photothermal Effect of PDA-ZnPc^+^ Nps

**Figure 3:**
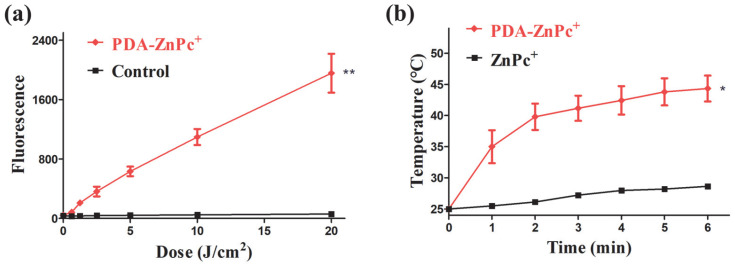
(**a**) The ROS generations of PDA-ZnPc^+^ Nps. (**b**) Photothermal heating curves of PDA-ZnPc^+^ Nps with laser irradiation time. The values are represented as means ± SEM, where ** *p* < 0.01, and * *p* < 0.05 vs. the control group.

In this study, 2,7-dichlorofluorescein diacetate (DCFH-DA), serving as a probe for detecting ROS, was employed to investigate the biological antitumor mechanisms of PDA-ZnPc^+^ Nps. The results of the fluorescence analysis of ROS generation are presented in Figure 3a. It is evident that the PDA-ZnPc^+^ Nps induced a significant increase in ROS release compared to the control group that did not receive PDA-ZnPc^+^ Nps treatment. Furthermore, the increment in ROS levels after illumination was substantial, indicating the potential of PDA-ZnPc^+^ Nps to exert a potent photodynamic therapy effect.

Additionally, we evaluated the photothermal effect of the PDA-ZnPc^+^ Nps by monitoring temperature changes under 808 nm laser irradiation (1.0 W/cm^2^). As depicted in Figure 3b, the PDA-ZnPc^+^ Nps exhibited a rapid temperature increase with the increase in the duration of irradiation, while the pure ZnPc^+^ solution showed only minimal temperature changes. The temperature of the PDA-ZnPc^+^ Nps reached approximately 45 °C after 5–6 min, highlighting the potential of PTT enabled by PDA-ZnPc^+^ Nps.

### 2.3. In Vitro Cytotoxicity of PDA-ZnPc^+^ Nps

The remarkable photothermal efficiency and ROS production of the PDA-ZnPc^+^ Nps prompted us to investigate their anticancer effects in vitro. The MTT assay was employed to investigate the cytotoxicity of PDA-ZnPc^+^ Nps against MCF-7 cells. Briefly, MCF-7 cells were incubated with various concentrations of PDA-ZnPc^+^ Nps (0.5, 1, and 2 mg/mL) to assess phototoxicity, photothermal toxicity, and the synergistic effects of PDT/PTT.

**Figure 4:**
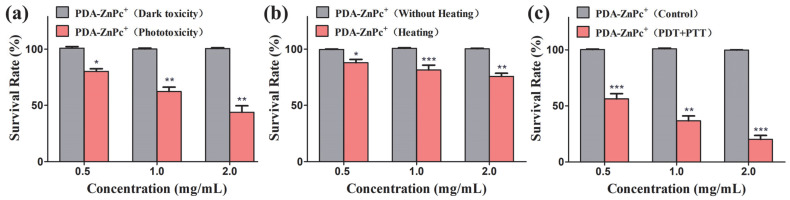
(**a**) Phototoxicity and dark toxicity of PDA-ZnPc^+^ Nps (0.5, 1, and 2 mg/mL) in MCF-7 cells. (**b**) Cytotoxicity of the MCF-7 cells cultured on PDA-ZnPc^+^ Nps (0.5, 1, and 2 mg/mL) with or without heating. (**c**) Cytotoxicity of the MCF-7 cells cultured on PDA-ZnPc^+^ Nps (0.5, 1, and 2 mg/mL) with or without synergistic PDT/PTT. The values are represented as means ± SEM; *** *p* < 0.001, ** *p* < 0.01, and * *p* < 0.05 vs. the control group.

The phototoxicity of PDA-ZnPc^+^ Nps on MCF-7 cells was measured and is shown in Figure 4a. These results indicate that the survival rate of MCF-7 cells significantly decreased with an increasing drug concentration. At a concentration of 2 mg/mL, cell viability was reduced to 43.8%, indicating the excellent PDT effect of PDA-ZnPc^+^ Nps. A similar dose-dependent pattern was observed for photothermal toxicity, with higher concentrations leading to greater cell death (Figure 4b). Furthermore, we explored the synergistic PDT/PTT effect of PDA-ZnPc^+^ Nps on MCF-7 cells (Figure 4c). Notably, at a concentration of 2 mg/mL, a significant level of toxicity was observed (79.9%), which was 1.42- and 3.29-fold higher than that of PDT or PTT treatment alone, respectively. These results highlight the excellent synergistic therapeutic effect of PDA-ZnPc^+^ Nps in MCF-7 cells when combined with PDT and PTT. Furthermore, nearly 100% of the MCF-7 cells survived after treatment with PDT, PTT, or synergistic PDT/PTT without illumination in all treatment groups (Figure 4), indicating the low dark toxicity and safety profile of PDA-ZnPc^+^ Nps. These findings further suggest that the synergistic PDT/PTT approach can achieve a superior therapeutic effect by combining the generation of ROS and thermal energy.

### 2.4. Apoptosis

**Figure 5:**
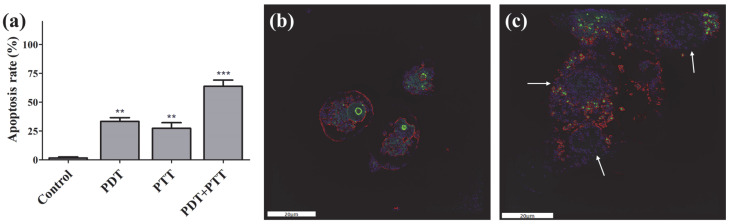
(**a**) Apoptosis rate of MCF-7 cells cultured on PDA-ZnPc^+^ Nps administered the control treatment, PDT, PTT, and synergistic PDT/PTT. (**b**) Fluorescence microscopy images of MCF-7 cells cultured on PDA-ZnPc^+^ Nps (without PDT or PTT). (**c**) Fluorescence microscopy images of MCF-7 cells cultured on PDA-ZnPc^+^ Nps (synergistic PDT/PTT). The values are represented as means ± SEM; *** *p* < 0.001 and ** *p* < 0.01 vs. the control group.

Apoptosis in MCF-7 cells induced by PDA-ZnPc^+^ Nps after treatment with PDT, PTT, and synergistic PDT/PTT was assessed using the Annexin V-FITC Apoptosis Detection Kit. MCF-7 cells were incubated with PDA-ZnPc^+^ Nps and irradiated with a 660 nm laser for PDT and/or an 808 nm laser for PTT. As depicted in Figure 5a, a negligible level of apoptosis was observed in the control MCF-7 cells cultured with PDA-ZnPc^+^ Nps but not treated with laser irradiation, indicating that the cultured PDA-ZnPc^+^ Nps alone (that did not undergo laser irradiation) did not induce significant toxicity. The PDT-treated group exhibited an apoptosis rate of 33.4%, while a relatively lower level of cytotoxicity (27.3%) was observed in the presence of the PTT-treated group. This finding suggests that both PDT and PTT individually induced apoptosis, which is consistent with the results of the in vitro cytotoxicity assay of PDA-ZnPc^+^ Nps. Importantly, the synergistic PDT/PTT group showed much stronger apoptosis (63.8%) upon 808 nm irradiation compared to PDT or PTT alone, thereby confirming a prominent synergistic effect.

### 2.5. Fluorescence Imaging

Fluorescence imaging was performed to investigate the cellular state of the MCF-7 cells after treatment with PDA-ZnPc^+^ Nps using a fluorescence microscope 3D Cell Explorer. PS fluorescence (red color) was observed in the cytoplasm, indicating the sub-cellular localization of ZnPc^+^. The treatment of the MCF-7 cells with PDA-ZnPc^+^ Nps without laser irradiation resulted in the cells maintaining a normal state (Figure 5b). Additionally, it was observed that the cells incubated with PDA-ZnPc^+^ Nps and subjected to both 650 nm and 808 nm laser irradiation exhibited significant levels of cell death, which were characterized by cell fragmentation and vacuolation (Figure 5c). These findings suggest that combination therapy is more effective than individual PDT or PTT with respect to inducing cell death.

### 2.6. In Vivo Combinational PTT/PDT

To evaluate the synergistic PDT/PTT efficacy of PDA-ZnPc^+^ Nps in vivo, we established an MCF-7 tumor-bearing Kunming murine model. The mice bearing MCF-7 tumors were divided into four groups (eight mice per group): a saline group (control), a PDA-ZnPc^+^ Nps with 680 nm laser irradiation group (PDT), a PDA-ZnPc^+^ Nps with 808 nm laser irradiation group (PTT), and a PDA-ZnPc^+^ Nps with both 680 and 808 nm laser irradiation group (PDT/PTT). The in vivo antitumor efficacies were evaluated by analyzing tumor growth inhibition and tumor weight.

**Figure 6:**
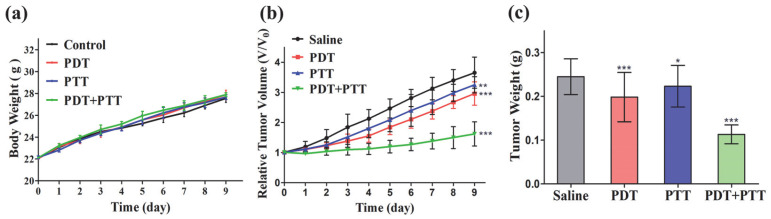
(**a**) Body weights of mice after various treatments over 9 days. (**b**) Tumor growth curves of mice under the different treatments administered. The tumor volumes were normalized to their initial sizes (V/V_0_). (**c**) Average weights of tumors collected from mice after treatments. The values are represented as means ± SEM; *** *p* < 0.001, ** *p* < 0.01, and * *p* < 0.05 vs. the control group.

As shown in Figure 6b, all the PDT, PTT, and synergistic PDT/PTT groups exhibited significant reductions in tumor growth rates compared to the control group after 9 days of treatment. In particular, on the 9th day, the relative tumor volume of the PDT group (2.96), the PTT group (3.25), and the synergistic PDT/PTT group (1.62) exhibited a 1.23-fold, 1.12-fold, and 2.25-fold reduction compared to the saline group (3.65), respectively. It should be noted that the synergistic PDT/PTT group showed better inhibition ratios compared to the PDT group (1.83-fold reduction) and PTT group (2.01-fold reduction), thus confirming the significant advantage and synergistic effect of combination therapy compared to monotherapy. Similar results were observed in the tumor weight analysis (Figure 6c), where the synergistic PDT/PTT group showed 42.9%, 49.3%, and 53.9% suppressions of tumor growth in the MCF-7-bearing mice compared to the PDT, PTT, and control groups, respectively. Importantly, the mice in each group presented increased body weights after treatment (Figure 6a), indicating that the therapy mediated by PDA-ZnPc^+^ Nps exerted no significant side effects in vivo.

### 2.7. In Vivo Safety Analysis of PDA-ZnPc^+^ Nps

**Figure 7:**
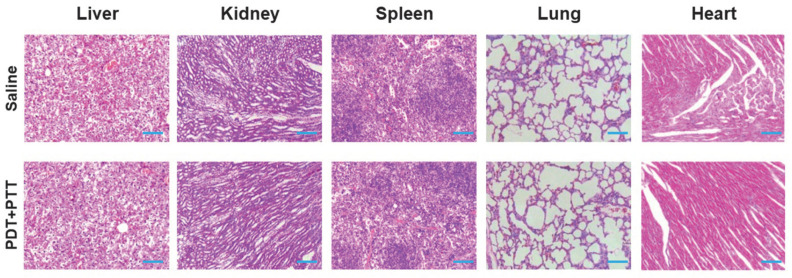
Representative H&E-stained images of major organs from the mice of the control (saline) and synergistic PDT/PTT treatment groups (Scale bar 75 μm).

Hematoxylin and eosin (H&E) staining assays were conducted to investigate the safety of PDA-ZnPc^+^ Nps in vivo. At the conclusion of the experiments, major organs, including the liver, kidneys, spleen, lungs, and heart, were harvested from the mice and subjected to H&E staining and examination using an inverted fluorescence microscope. As depicted in Figure 7, no significant histopathological abnormalities or lesions were observed in these organs in the synergistic PDT/PTT group compared to the control group (Saline). These findings suggest the negligible toxic side effects of the synergistic therapy mediated by PDA-ZnPc^+^ Nps.

## 3. Discussion

In this study, we have presented a novel approach to achieving synergistic PDT and PTT by developing polydopamine-based nanoparticles (PDA-ZnPc^+^ Nps) loaded with an efficient photosensitizer ZnPc^+^. Our work addresses the issue of dark toxicity associated with photosensitizers, which limits their clinical application, particularly in achieving effective drug concentrations and dosages for treatments [31,35]. By incorporating the photothermal effect of polydopamine (PDA), the PDA-ZnPc^+^ Nps developed in this study not only enable PDT but also facilitate PTT, further enhancing the therapeutic potential of the nanoparticles. Moreover, the nanoparticles can leverage the enhanced permeability and retention (EPR) effect, thus improving their ability to target tumor cells [36,37].

The utilization of PDA as a nanocarrier offers several advantages. Firstly, PDA exhibits remarkable biocompatibility and has been demonstrated to be safe and stable in vivo and does not induce toxic side effects on the body [38,39,40]. This ensures the overall safety of PDA-ZnPc^+^ Nps as a therapeutic agent. Additionally, PDA provides efficient and stable loading of drug molecules, making it an ideal carrier with a strong affinity for tumor cells. In this study, we synthesized ZnPc^+^ with 12 positive charges, enabling its stable adsorption onto PDA through interaction, resulting in efficient drug loading.

The synergistic PDT/PTT treatment achieved using the PDA-ZnPc^+^ Nps not only effectively kills tumor cells but also allows for the use of photosensitizers within a safe concentration and dosage range, thereby striking a balance between treatment efficacy and safety. Firstly, PDA-ZnPc^+^ Nps induce cytotoxicity mainly by photothermal and photodynamic means. Both PDT and PTT treatments require illumination with the help of special light sources, resulting in the generation of ROSs and thermal effects that kill tumor cells. Targeted illumination of tumor tissue or cells significantly reduces the effects of photodynamic and photothermal effects on normal tissues and cells. In addition, the dark toxicity and cytotoxicity of PDA-ZnPc^+^ Nps demonstrated that PDA-ZnPc^+^ Nps has little toxic effect on cells without illumination. Secondly, in the in vivo experiments, we analyzed the sections of several important tissues and organs, including the heart, liver, spleen, kidneys, and lungs, of the mice after treatments. The results showed that the synergistic treatment carried out using the PDA-ZnPc^+^ Nps had no significant effect on the important organs of the mice, and the weight growth trend of the experimental and control group was consistent, which proved that therapy had little effect on normal tissues and cells. It is worth noting that the current PDT light source equipment is continually being upgraded and developed, and the research and application of some endoscopes and built-in light sources are expected to promote the application of PDT in the treatment of deep tumors. Our findings hold promise for the application of photosensitizers and nanomedicine, providing a valuable reference for reducing toxic side effects while improving treatment outcomes.

## 4. Materials and Methods

### 4.1. Chemicals and Cell Lines

Chemicals used in this study were purchased from Sigma-Aldrich (St Louis, MO, USA) or Sinopharm Chemical Reagent Co., Ltd. (Shanghai, China) and were of analytical grade. The human breast adenocarcinoma cell line MCF-7 was obtained from the Shanghai Institute of Cell Biology, Chinese Academy of Sciences, Shanghai, China. MCF-7 cells were cultured in Dulbecco’s modified Eagle’s medium (DMEM) supplemented with 10% fetal calf serum (FCS) and 1% penicillin-streptomycin at 37 °C under humidified air with 5% CO_2_. To establish the MCF-7 tumor-bearing mouse model, MCF-7 cells were preserved in liquid nitrogen and passaged weekly through Kunming mice in the form of ascites to enhance cell viability.

### 4.2. Preparation of ZnPc(4TAP)^12+^ (ZnPc^+^)

The compound ZnPc(4TAP)^12+^ was synthesized as shown in Figure S1 using a slightly modified previously reported method [34]. Briefly, 83 mg of synthetic precursor ZnPc (4TAP) (0.05 mmol) dissolved in 50 mL of DMF was reacted with 43 mL of CH_3_I (0.67 mmol) for 6 h at room temperature to obtain cationic ZnPc. The reaction mixture was concentrated to 5 mL under reduced pressure, and the product was precipitated by adding diethyl ether. The precipitates were collected via centrifugation, dissolved in methanol, filtered to remove insoluble impurities, and precipitated again with diethyl ether. The product was centrifuged, dried under vacuum, and obtained as ZnPc(4TAP)^12+^, which was soluble in water (>10 mg/mL).

### 4.3. Synthesis of PDA

To synthesize PDA nanoparticles with an average diameter of 170 nm, a mixture of 90 mL of deionized water, 2 mL of ammonia aqueous solution (NH_4_OH, 28–30%), and 40 mL of ethanol was stirred at 30 °C for 1 h. Then, 500 mg of dopamine hydrochloride was dissolved in 10 mL of deionized water and injected into the mixture solution. The color of the solution changed to pale yellow and dark brown, indicating the polymerization reaction of dopamine hydrochloride. The reaction was allowed to proceed for 24 h. PDA nanoparticles were obtained via centrifugation, washed with deionized water until the supernatant became colorless, and then re-dispersed in deionized water.

### 4.4. Synthesis of PDA-ZnPc(4TAP)^12+^ Nanoparticles (PDA-ZnPc^+^ Nps)

To load ZnPc^+^ onto the PDA nanoparticles, a PDA solution (2 mg/mL) was mixed with an excess amount of ZnPc^+^ and stirred for 2 h. Unbound ZnPc^+^ was removed via centrifugal filtration. The resulting PDA-ZnPc^+^ was dispersed in water, yielding a dark brown solution similar to the PDA solution. The PDA-ZnPc^+^ Nps were stored at 4 °C for subsequent experiments. The degree of loading (DOL) of ZnPc^+^ in PDA-ZnPc^+^ Nps was determined based on the specific absorbance at 690 nm. The amount of ZnPc^+^ loaded onto the PDA-ZnPc^+^ Nps (mg) was calculated using standard curves derived from the absorbance of pure ZnPc^+^. The DOL was calculated using the following equation: DOL (%) = (weight of ZnPc^+^)/(weight of PDA-ZnPc^+^ Nps) × 100%.

### 4.5. Characterization of PDA-ZnPc^+^ Nps

The morphology of the samples was examined using a JSM 6700F scanning electron microscope. The UV-vis absorption spectrum of PDA-ZnPc^+^ Nps in DI water was measured using a Synergy 4 multi-mode microplate reader (BioTek Instruments, Winooski, VT, USA). The size distribution and zeta potentials of PDA-ZnPc^+^ Nps were determined by taking dynamic light scattering (DLS) measurements (Nano ZS90, Malvern Instruments, Worcestershire, UK). The loading capacity of ZnPc^+^ was evaluated based on the interaction and π–π stacking. PDA solutions (1 mg/mL) were mixed with excess ZnPc^+^ for varying durations (1, 2, 3, or 4 h), and unbound ZnPc^+^ was removed via centrifugal filtration. The stability of PDA-ZnPc^+^ Nps was assessed by monitoring changes in size over a period of 7 days in saline solution at room temperature. The dispersibility of PDA-ZnPc^+^ Nps was examined by dispersing them in water, saline, and PBS at room temperature. The data curves were fitted to exponential functions using Origin and Prism-5 software.

### 4.6. ROS and Photothermal Effect of PDA-ZnPc^+^ Nps

ROS are among the main causes of tumor cell death in PDT treatment. DCFH-DA was used for ROS detection in this study to investigate the mechanisms of PDT induced by PDA-ZnPc^+^ Nps. Briefly, PDA-ZnPc^+^ Nps were plated onto plates (2 mg/mL, 200 μL/well), and 10 μM of DCFH-DA was incubated in 10 min before illumination with LED light source at different light doses (0.625, 1.25, 2.5, 5, 10, and 20 J/cm^2^). Then, the fluorescence of DCF-DA at 528 nm was determined after illumination. The wells without PDA-ZnPc^+^ Nps served as blanks. The photothermal effect induced by PDA-ZnPc^+^ Nps was investigated using an 808 nm laser (1.0 W/cm^2^, Changchun New Industry Photoelectric Technology Co., Ltd., Changchun, China). The temperatures were recorded every minute for a total of 6 min, and pure ZnPc^+^ solution was used as a control. Temperature changes were measured using a DAE-905k thermometer (SENDAE, Guangzhou, China) at 1 min intervals for a total of 6 min. Experiments were performed in triplicate.

### 4.7. In Vitro PDT, PTT, and Synergistic PDT/PTT

The viabilities of MCF-7 cells upon treatment with PDA-ZnPc^+^ Nps in the PDT, PTT, or synergistic PDT/PTT treatments were evaluated using standard methyl thiazolyltetrazolium (MTT) assays, which assess cell viability based on the reduction of yellow MTT to insoluble purple formazan. MCF-7 cells at a density of 5 × 10^4^ cells/mL were collected and plated in 96-multiwell plates with culture medium (200 μL/well).

For PDT treatment, the cells were incubated with PDA-ZnPc^+^ Nps at various concentrations (0.5, 1, and 2 mg/mL) for 2 h, followed by 2 min of illumination at doses of 5 J/cm^2^ using a planar LED light source (660 nm). Dark toxicity experiments of PDA-ZnPc^+^ Nps on MCF-7 cells were also conducted.

For PTT treatment, the cells were incubated with PDA-ZnPc^+^ Nps at various concentrations (0.5, 1, and 2 mg/mL) for 2 h, followed by 5 min of illumination using an 808 nm laser. Control experiments without the heating of PDA-ZnPc^+^ Nps on the MCF-7 cells were also carried out.

For synergistic PDT/PTT treatment, the cells were incubated with PDA-ZnPc^+^ Nps at various concentrations (0.5, 1, and 2 mg/mL) for 2 h, followed by 2 min of illumination at doses of 5 J/cm^2^ using a LED light source (660 nm) and 5 min of illumination using an 808 nm laser. The survival rate of the cells that were not treated was also determined for comparison.

After 24 h of incubation, 100 μL MTT solution (0.5 mg/mL) in DMEM was added to the cell plate and allowed to continue culturing for 3 h. The insoluble purple formic acid product was dissolved in 100 μL of dimethyl sulfoxide (DMSO) to obtain a colored solution. Its absorbance was measured at 570 nm using a Synergy 4 multi-mode microplate reader. Each experiment was repeated three times, with three replicates per concentration.

### 4.8. Apoptosis Detection

Apoptosis of MCF-7 cells induced by PDA-ZnPc^+^ Nps after PDT, PTT, and PDT/PTT was measured and analyzed using an Annexin V-FITC apoptosis assay kit (Beyotime Biotechnology, Shanghai, China). Annexin V binds to fluorescent dye FITC and membrane phospholipid phosphatidylserine as a sensitive probe for apoptosis analysis via flow cytometry. MCF-7 cells were inoculated in a 6-well plate at a density of 5 × 10^4^ cells/mL (2 mL/well). After 16 h of incubation at 37 °C for cell adhesion, the cells were treated with PDA-ZnPc^+^ Nps at a concentration of 1 mg/mL for 1 h. After being carefully washed with PBS, the cells were subjected to 2 min of illumination (using a 660 nm LED light source) and 5 min of illumination (using an 808 nm laser). Cells that were left untreated served as a control.

### 4.9. Fluorescence Imaging

Fluorescence imaging of MCF-7 cells treated using PDA-ZnPc^+^ Nps or left untreated were investigated to observe their cell states. MCF-7 cells were planted in 6-well plates with a medium density of 5 × 10^4^ at 2 mL per well and incubated overnight. Next day, the MCF-7 cells were further incubated with 1 mg/mL of PDA-ZnPc^+^ Nps for 1 h, while the PDT/PTT group was further treated with 2 min (660 nm LED light source) and 5 min of illumination (808 nm laser). Then, the plates were carefully washed thrice with PBS to remove the unabsorbed compounds. The fluorescence imaging of MCF-7 cells with or without illumination was performed using a fluorescence microscope (3D Cell Explorer).

### 4.10. Establishment of MCF-7 Tumor-Bearing Mouse Model

Kunming mice (4 weeks old, weighing 20–21 g, and purchased from Shanghai SLAC Laboratory Animal Co. Ltd., Shanghai, China) were maintained and handled following the recommendations of the institutional animal care and use committee (IACUC). The mice had free access to water and food throughout the experimental period. To establish the MCF-7 tumor model using the Kunming mice, ascites containing MCF-7 cells were obtained from the abdominal cavities of the tumor-bearing mice after inoculation on day 5. The ascites was diluted with sterile saline to 1.0 × 10^7^ cells/mL, and 0.2 mL of it was subcutaneously inoculated on the right side of the back of each mouse. The treatment trial was initiated when the tumor length (the longest diameter of the tumor) reached 5–7 mm and the width (the diameter perpendicular to the length) reached 4–6 mm.

### 4.11. In Vivo Combinational PTT/PDT

The antitumor activity of PDA-ZnPc^+^ Nps against MCF-7 tumor models implanted in Kunming mice was evaluated through growth inhibition analysis. MCF-7 tumor-bearing mice (established as described above) were randomly divided into four groups, each consisting of eight mice: control, PDT, PTT, and synergistic PDT/PTT. The in vivo assay began with similar average initial tumor sizes (~50 mm^3^) and body weights (~22 g) in each group. The mice in the PDT, PTT, and synergistic PDT/PTT groups were administered PDA-ZnPc^+^ Nps via the caudal vein, while the control group was injected intravenously with an equivalent volume of 0.9% saline. After 8 h, the MCF-7 tumors in the PDT group were treated with 680 nm of light illumination using a LumaCare non-coherent light source for 10 min, resulting in a total light fluence of 100 J/cm^2^. The MCF-7 tumors in the PTT group were irradiated with an 808 nm laser at a power density of 1.0 W/cm^2^ for 10 min. The MCF-7 tumors in the synergistic PDT/PTT group were treated with both 680 nm and 808 nm laser illumination for 10 min each. The weights of the mice were monitored every day, and the tumor sizes were calculated with a caliper during the experiment. The calculation formula was ellipsoid volume formula (W^2^ × L) × π/6, where W denotes tumor width and L denotes tumor length. At the end of the treatments, the mice were killed, and the MCF-7 tumors were carefully dissected and weighed. Tumor growth inhibition and tumor gravimetric analysis were used to evaluate the efficacy of treatments of the MCF-7 tumors in the mice.

### 4.12. H&E Staining Assay

The hematoxylin and eosin (H&E) staining assay was carried out to investigate the biosafety of PDA-ZnPc^+^ Nps in vivo by examining the tissue morphology of major organs. After the completion of the in vivo PDT/PTT antitumor experiments, the major organs, including the heart, liver, spleen, lungs, and kidneys, were retrieved from the mice and fixed in 10% formalin. The samples were then embedded in paraffin and sectioned into 4 mm thick sections, which were stained with H&E. The stained sections were examined under an inverted fluorescence microscope to assess any pathological abnormalities or lesions.

### 4.13. Statistical Analysis

All data presented in this study represent group means and standard errors of the mean (SEM). Statistical analysis was performed using two-way analysis of variance (ANOVA). Differences were considered statistically significant at the 95% confidence level (*p* < 0.05).

## 5. Conclusions

In conclusion, we have developed and investigated a dual-functional antitumor compound, PDA-ZnPc^+^ Nps, both in vitro and in vivo. By utilizing the unique chemical structure and photothermal effect of PDA, we successfully attached the photosensitizer ZnPc^+^ to PDA Nps, resulting in synergistic PDT/PTT anticancer actions. The PDA-ZnPc^+^ Nps demonstrated high ROS production levels and photothermal efficiency, leading to cell apoptosis, cell destruction, and vacuolation, ultimately improving the efficacy of PDT. The synergistic PDT/PTT exhibited superior anticancer efficacy compared to individual treatments in vitro, highlighting the synergistic effect of the PDA carrier and ZnPc^+^ photosensitizer. In addition, PDA-ZnPc^+^ Nps showed significant synergistic antitumor effects in vivo in the MCF-7 tumor-bearing mouse models without presenting significant systemic damage. The mice presented good biocompatibility and no observable toxic side effects, suggesting that photosensitizer-functionalized PDA-based nanomaterials are promising for the design of multifunctional anticancer drugs through synergistic photodynamic and photothermal therapy.

## Data Availability

Not applicable.

## Supplementary Materials

The following supporting information can be downloaded at: https://www.mdpi.com/article/10.3390/molecules28155874/s1. Figure S1. Synthesis of ZnPc(4TAP)12+ (ZnPc+). Figure S2. Size distributions changes of PDA-ZnPc+ Nps during 7 days. Figure S3. Photographs of PDA-ZnPc+ Nps in water, saline and PBS during 24 h.
