# Supplementary material for: A Photosensitizer-Loaded Polydopamine Nanomedicine Agent for Synergistic Photodynamic and Photothermal Therapy
Source: Molecules. 2023 Aug 4;28(15):5874. doi: 10.3390/molecules28155874 (PMC10420639; doi:10.3390/molecules28155874)
Supplement: Supplementary file 1 [file molecules-28-05874-s001.zip › molecules-2505421-supplementary.pdf]

<u>Contents</u>

1. **Figure S1**. Synthesis of $ZnPc(4TAP)^{12+}$ ($ZnPc^{+}$).

2. **Figure S2**. Size distributions changes of PDA-$ZnPc^{+}$ Nps during 7 days.

3. **Figure S3**. Photographs of PDA-$ZnPc^{+}$ Nps in water, saline and PBS during 24 hours.

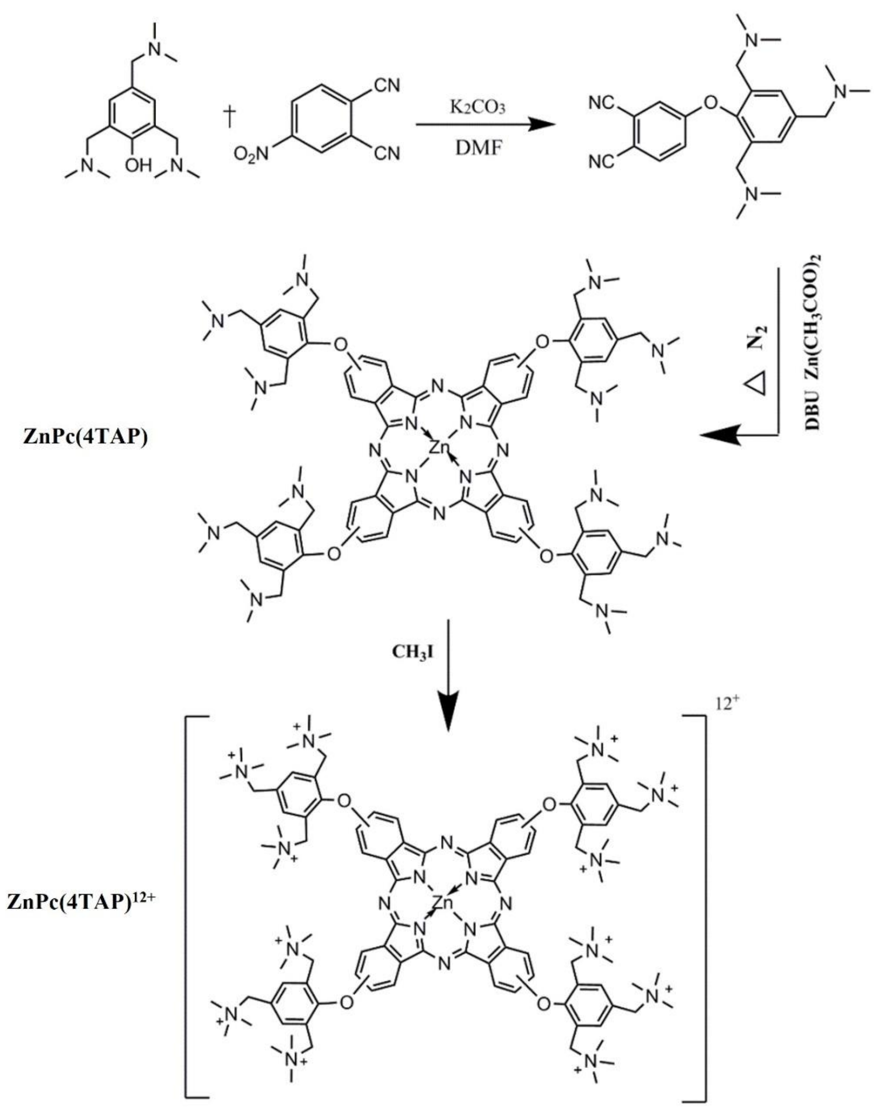

Figure S1. Synthesis of ZnPc(4TAP)$^{12+}$ (ZnPc^{+}).

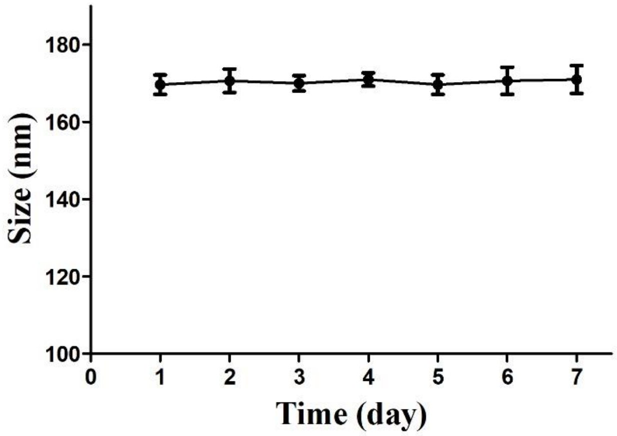

Figure S2. Size distributions changes of PDA-$ZnPc^+$ Nps during 7 days.

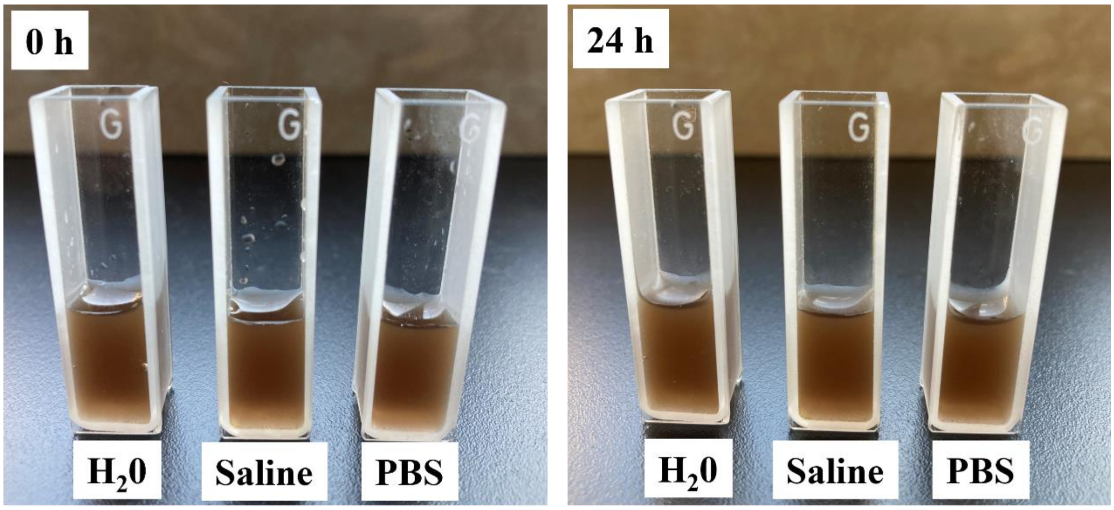

Figure S3. Photographs of PDA-$ZnPc^+$ Nps in water, saline and PBS during 24 hours.
